# Phenotypic and Genetic Heterogeneity of a Pakistani Cohort of 15 Consanguineous Families Segregating Variants in Leber Congenital Amaurosis-Associated Genes

**DOI:** 10.3390/genes15121646

**Published:** 2024-12-21

**Authors:** Zainab Akhtar, Sumaira Altaf, Yumei Li, Sana Bibi, Jamal Shah, Kiran Afshan, Meng Wang, Hafiz Muhammad Jafar Hussain, Nadeem Qureshi, Rui Chen, Sabika Firasat

**Affiliations:** 1 Department of Zoology, Faculty of Biological Sciences, Quaid-i-Azam University, University Road, Islamabad 45320, Pakistan; zainabakhtar@bs.qau.edu.pk (Z.A.); kafshan@qau.edu.pk (K.A.); 2 Department of Pediatric Ophthalmology and Strabismus, Al-Shifa Trust Eye Hospital, Jhelum Road, Rawalpindi 46000, Pakistan; 3 Department of Ophthalmology, Center for Translational Vision Research, Irvine School of Medicine, University of California, Irvine, CA 92697, USA; yumeil3@hs.uci.edu (Y.L.); mengw19@hs.uci.edu (M.W.); 4 Department of Molecular and Human Genetics, Baylor College of Medicine, Houston, TX 77030, USA; hafizmuhammadjafar.hussain@bcm.edu

**Keywords:** Leber congenital amaurosis, childhood blindness, autosomal recessive, genetic heterogeneity

## Abstract

Background: Leber congenital amaurosis (LCA) is a congenital onset severe form of inherited retinal dystrophy (IRD) and a common cause of pediatric blindness. Disease-causing variants in at least 14 genes are reported to predispose LCA phenotype. LCA is inherited as an autosomal recessive disease. It can be an isolated eye disorder or as part of a syndrome, such as Senior Loken or Joubert syndrome. Sequencing studies from consanguineous populations have proven useful for novel variants identification; thus, the present study aimed to explore the genetic heterogeneity of 15 consanguineous Pakistani families, each segregating a severe IRD phenotype using targeted next generation sequencing. Methods: This study enrolled 15 consanguineous families, each with multiple affected cases of retinal dystrophy phenotype. DNA was extracted from blood samples. Targeted panel sequencing of 344 known genes for IRDs was performed, followed by Sanger sequencing for segregation analysis. Results: Data analysis revealed a total of eight reported (c.316C>T and c.506G>A in *RDH12*; c.864dup and c.1012C>T in *SPATA7,* as well as c.1459T>C, c.1062_1068del, c.1495+1G>A, c.998G>A in the *CRB1*, *LCA5*, *TULP1,* and *IFT140* genes, respectively) and four novel homozygous (c.720+1G>T in *LCA5*, c.196G>C in *LRAT*, c.620_625del in *PRPH2,* and c.3411_3414del in *CRB1*) variants segregating with disease phenotype in each respective family. Furthermore, a novel heterozygous variant of *CRB1* gene, i.e., c.1935delC in compound heterozygous condition was found segregating with disease phenotype in one large family with multiple consanguinity loops. Conclusion: Comprehensive molecular diagnosis of 15 consanguineous Pakistani families led to the identification of a total of 5 novel variants contributing to genetic heterogeneity of LCA-associated genes and helped to provide genetic counseling to the affected families.

## 1. Introduction

Leber congenital amaurosis (LCA; MIM 204000) is a genetically and clinically heterogeneous inherited retinal dystrophy (IRD) that accounts for 5% of all IRDs and 20% of childhood blindness [1,2]. Clinical presentation of LCA includes severe visual impairment, nystagmus, amaurotic pupils, and electroretinogram (ERG) abnormalities within the first year of life [1,3]. The fundus appearance in LCA patients varies, either appearing unremarkable at early stages, or exhibiting pigmentary changes, i.e., the optic disc pallor and retinal pigment clumping that usually appear in older individuals [3]. Other ocular findings that may or may not be present in LCA patients include photophobia, early-onset macular atrophy, night blindness, hyperopia, peripheral retinal abnormalities, keratoconus, and cataracts [1,3]. LCA is mostly non-syndromic; however, some causative variants have systemic involvement [2,4].

LCA is predominantly inherited in an autosomal recessive manner; however, there are a few reports of autosomal dominant inheritance as well [2,5]. To date, disease-causing variants in 27 genes have been identified to cause LCA phenotypes (RetNet Retinal Information Network) [6]. The LCA proteins are typically involved in retinal development (CRX), various retinal pathways such as photoreceptor morphogenesis (CRB1, GDF6), retinal pigment epithelium phagocytosis (MERTK), the retinoid cycle (RDH12, LRAT), vitamin A metabolism (RPE65), protein multimerization (NMNAT1), phototransduction (GUCY2D, AIPL1, KCNJ13, CABP4), guanine synthesis (IMPDH1) and intraphotoreceptor ciliary transport (SPATA7, LCA5, RPGRIP1, TULP1, CLUAP1, IQCB1, IFT140, ALMS1, CEP290) [4,6,7]. As different location and pathophysiological mechanisms have been found for each of the protein encoded by LCA-associated genes, thus pathogenic variant/s causing the dysfunction or absence of their encoded protein/s compromise the normal visual cycle [3,6]. The most frequent genetic causes of LCA include the *CEP290* (15%), *GUCY2D* (12%), *CRB1* (10%), and *RPE65* (8%) genes, which account for 70–80% of known cases [4,6].

Due to genetic diversity, early onset, and the severe visual impairment caused by LCA, determination of the molecular basis of the disease is important to obtain a definitive diagnosis for patient management, genetic counseling and translational research. The present study aimed to report phenotypic and genetic heterogeneity of a Pakistani cohort of 15 consanguineous families, each with multiple affected individuals segregating severe retinal dystrophy phenotypes and variants in LCA-associated genes. We performed panel sequencing of these families to analyze the exons of 344 known inherited retinal disease genes. Our results highlight CRB1 as the most frequently mutated LCA gene in our study cohort.

## 2. Materials and Methods

### 2.1. Ethical Approval and Enrollment of Families

The Bio-Ethical review Committee of Faculty of Biological Sciences, Quaid-i-Azam University Islamabad, Pakistan and Ethical Review Committee, Al-Shifa Trust, Rawalpindi, Pakistan approved this study. Fifteen families, each segregating a severe form of Leber congenital amaurosis (LCA), were clinically evaluated by ophthalmologists at Al-Shifa trust eye hospital, Rawalpindi, Pakistan and were recruited following the principles of the Declaration of Helsinki for molecular genetic analysis. Clinical assessments include detailed family and medical history, physical examination, fundoscopy, slit lamp exam and visual acuity testing. In total, 3–5 mL of venous blood was collected after signed informed consent from the proband as well as other available members of each enrolled family. Genomic DNA extraction and quantification was performed at Department of Zoology, Quaid-i-Azam University, Islamabad, Pakistan as per our previously reported method [7].

### 2.2. Targeted Exome Sequencing and Bioinformatic Analysis

Capture panel sequencing was performed using genomic DNA of two affected individuals of each enrolled family at Baylor College of Medicine, Houston, TX, USA. Exome-enriched genomic libraries were prepared using the KAPA HyperPrep Kit (Roche, Basel, Switzerland) following the manufacturer’s protocol, then they were pooled together for targeted enrichment of a panel of 344 known and candidate inherited retinal diseases related genes (as described in our previous study [7] with the SureSelect Target Enrichment System for the Illumina Platform (Agilent, Santa Clara, CA, USA) [8]. Captured DNA was quantified and sequenced using a Novaseq 6000 (Illumina, San Diego, CA, USA). Variant calling, data alignment, and filtration were performed at the Functional Genomics Core at Baylor College of Medicine, USA, as described in our previous studies [7,8]. Variants passing the filtering steps were evaluated as suggested by the American College of Medical Genetics and Genomics (ACMG) guidelines for their interpretation (Supplementary Table S1). Previously reported pathogenic variants were detected and searched through HGMD, ClinVar (https://www.ncbi.nlm.nih.gov/clinvar/, accessed on 30 July 2024) and LOVD (http://www.lovd.nl, accessed on 31 July 2024) databases. Novel variants were evaluated for their potential impact on protein function using multiple in silico tools. Missense, frameshift, and splice site variants were classified as likely loss-of-function alleles. Missense variants were evaluated based on sequence conservation and in silico predictions (Supplementary Table S1). All novel and previously reported likely pathogenic variants were curated based on the data of the present study as per ACMG guidelines.

### 2.3. Sanger’s Sequencing and Segregation Test

For segregation of identified variants within a family, primers were designed using the Primer3 web resource (http://bioinfo.ut.ee/primer3-0.4.0/, accessed on 15 April 2024) to perform Sanger’s sequencing. Sanger’s sequencing was performed for the proband, and other affected and unaffected members of each family, based on the availability of DNA samples.

## 3. Results

### 3.1. Clinical Phenotype

**Figure 1:**
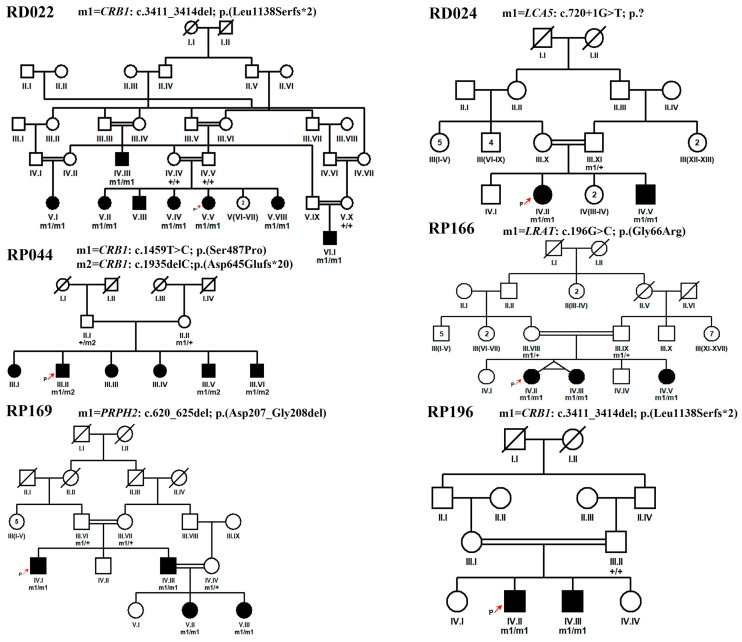
Pedigrees of LCA families with novel variants were identified in this study. Squares and circles symbolize males and females, respectively. Clear symbols indicate unaffected family members while filled symbols indicate patients. Consanguineous marriages are indicated by double lines. The symbol labeled with a red arrow in each pedigree highlights the proband.

In the current study, 15 multigenerational consanguineous families, each segregating severe early-onset inherited retinal dystrophy suggestive of the Leber congenital amaurosis (LCA) phenotype, were enrolled through ophthalmologists at Al-Shifa trust eye hospital, Rawalpindi, Pakistan. Among these families, seven families (RD022, RP002, RP028, RP169, RP184, RP194 and RP196) were Punjabi, six (RD023, RD036, RP044, RP159, RP166 and RP187) were Pashtun, and two families, i.e., RD024 and RD027 were Saraiki and Pothohari, respectively. The clinical data of the affected individuals of these families at the time of enrollment are detailed in Table 1. Detailed interviews with the elders of each family revealed ethnicity, onset ages, symptoms, and a complete family history for drawing pedigrees (Figure 1, Supplementary Figures S1–S4).

The probands of all families (except for RD023) had congenital onset of nyctalopia (Table 1). All the affected members of the families RD022, RD023, RD036, RP159, and RP196, and a few from the other families RP028 (IV.III), RP184 (IV.I), RP169 (IV.I), and RP194 (IV.III), had photophobia. Refractive error with hyperopia was present in the proband of RD022, RD023 and RP184 myopia in RP027, RP166, and RP194 and astigmatism was detected in RD036 proband. All the patients of the families RD023, RD024, RD036, RP159, and RP184 had nystagmus eyes which were also present in some affected cases of other families, including RD022 (VI.I), RD027 (III.II), RP196 (V.II) and RP196 (IV.II) (Figure 1 and Supplementary Figures S1–S4). Only an affected case of RD022 (V.IV) was observed with keratoconus. Other ocular and systemic phenotypic features recorded in some of the LCA patients in this study were strabismus, squint, hearing loss, intellectual disability, hypertension, heart issue, kidney problems, and teeth and bone deformities (Table 1). Hearing loss was present in only two cases, i.e., RD023 (IV.III) and RD027 (III.V). Patients from families, i.e., RD027, (III.V), RP166 (IV.III), RP184 (IV.VI) were intellectually disabled (Table 1).

**Figure 2:**
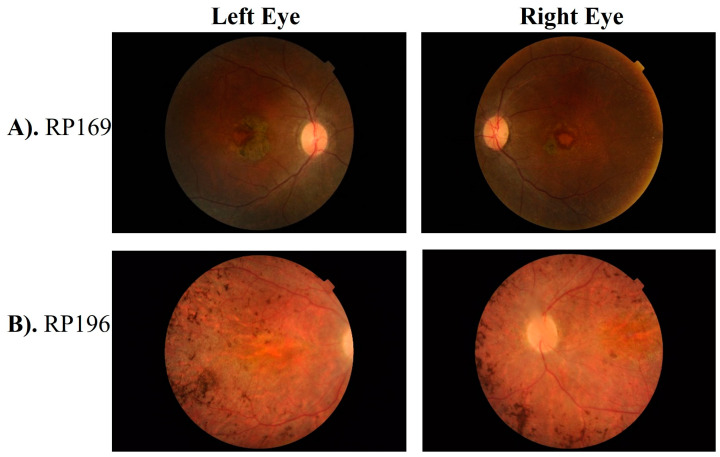
Fundus photograph of left and right eye of proband of family RP169 (**A**) and RP196 (**B**) showing waxy bone spicule with attenuated vessels, maculopathy, and pigmentary changes with pigmented clumps at retinal pigment epithelium, which are characteristic of LCA phenotype.

Fundus examination of probands of families RD022, RD023, RD027, RD036, RP159, RP166, RP169, RP194, and RP196 showed waxy bone spicule with vascular attenuation. Other fundus features including atrophic maculopathy, dull atrophic macula, dull foveal reflexes, and cellophane maculopathy were seen in RD022, RD027, RD036, and RP166, respectively. Representative fundus photographs are shown in Figure 2 and Supplementary Figure S5.

### 3.2. Genetic Screening

For the genetic screening of 15 consanguineous IRD-affected families, capture panel sequencing followed by Sanger sequencing validation was performed. Autosomal recessive inheritance was assumed based on the family pedigree. Consistently, putative homozygous mutations were identified, containing thirteen variants from eight genes, including one in frame, one stop gain, two splice sites, four frameshifts and four missense variants (Table 2).

Variants from four genes, the *CRB1*, *LCA5*, *RDH12,* and *SPATA7* genes, were found in more than one family. Among them, *CRB1* is the most frequently mutated gene in our study cohort, observed in five families (Table 2). While eight variants have been previously reported, five variants are newly identified by the current study. Autosomal recessive homozygous variants were segregating in all families, except for one with autosomal recessive compound heterozygous variants (RP044). Family trees with identified novel and reported variants with protein changes are given in Figure 1 and Supplementary Figures S1–S4, respectively. All the identified variants were classified as per the ACMG guidelines [9], and their details are provided in Table 2 and Table 3.

Sequence variant nomenclature was obtained according to the guidelines of the Human Genome Variation Society (HGVS) by using Mutalyzer (https://mutalyzer.nl/, accessed on 16 August 2024). The positions of variants are according to GRCh37/hg19 reference genome assembly.

**Figure 3:**
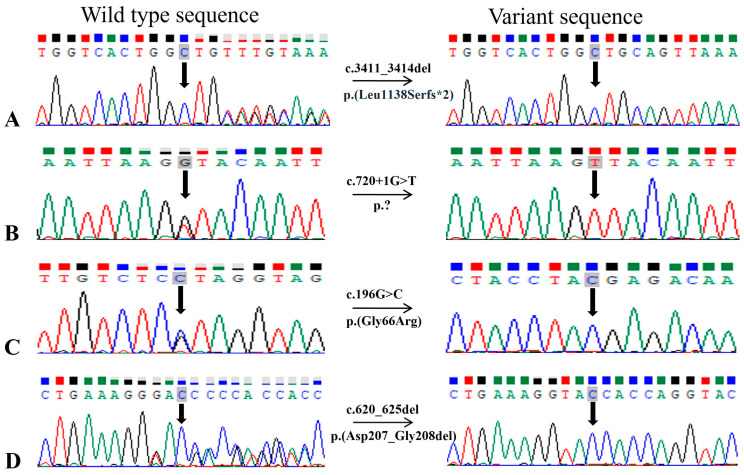
Sequence chromatograms for homozygous novel variants were identified in this study. The wild-type or carrier sequence is shown on the left and the mutated sequence is shown on the right side. (**A**) Deletion of 4 nucleotides (TGTT) in *CRB1* gene in family RD022 and RP196. The sequence TGTT, present in normal individuals of the family but deleted in affected ones. (**B**) Novel splice site variant in *LCA5* gene in RD024. (**C**) Novel missense variant in *LRAT* gene in family RP166. (**D**) Novel in frame variant in *PRPH2* gene in family RP169.

Multiple *CRB1* gene variants segregating with disease phenotype are found in five unrelated families (Figure 1 and Supplementary Figure S1). A novel frameshift variant c.3411_3414del causing p. (Leu1138Serfs*2) was identified as pathogenic and segregating with LCA/EOSRD phenotypes in two consanguineous Punjabi families RD022 and RP196 with eight and two affected cases, respectively (Figure 1). Segregation of this variant with the disease was confirmed by genotyping seven affected and three unaffected family members of RD022 and two affected and one unaffected member of RP196, further supporting pathogenicity (Figure 3A, Table 3). Detailed clinical examination of probands revealed additional symptoms including photophobia, color vision problems, hyperopia, and oculo-digital sign in RD022 (Table 1) and photophobia, nystagmus, and myopia in RP196 (Table 1). The patients of family RD022 had additional symptoms including nystagmus in individual VI. I, keratoconus in V.IV and strabismus in the IV.III affected case. Variant p. (Leu1138Serfs*2) is likely to be pathogenic, as early termination removes approximately 20% of the protein [10,11,12].

A previously reported missense variant, i.e., c.1459T>C of *CRB1* gene was found segregating as an autosomal recessive homozygous allele in two Pashtun families, RD023 and RP159. This variant substitute serine for proline at 487 position in protein, i.e., p. (Ser487Pro) (Supplementary Figures S1 and S6A). Furthermore, validation of the segregation of this variant in six homozygous-affected cases belonging to two families (RD023 and RP159) in this study label this variant as pathogenic instead of, different from the previously reported likely pathogenic status in ClinVar (Table 2 and Table 3). The same variant was observed in RP044 (Pashtun) as a heterozygous allele segregating in compound heterozygous form with another novel heterozygous variant c.1935delC; p. (Asp645Glufs*20) in the same gene (Figure 1). Both variants were segregating with disease phenotype in three affected and two unaffected individuals of family RP044. The nummular pigment clumps in the retina, characteristic of *CRB1* mutations, were found in the patients of families RP196 and RD023 (Figure 2B and Supplementary Figure S5A).

PVS1: Pathogenic very strong [null variant (nonsense, frameshift, canonical ± 1 or 2 splice sites, initiation codon, single or multiexon deletion) in a gene; here, LOF is a known mechanism of disease]. PM2: Pathogenic moderate 2 [Absent from controls (or at extremely low frequency if recessive) in Exome Sequencing Project, 1000 Genomes Project, or Exome Aggregation Consortium]. PM4: [Protein length changes as a result of in-frame deletions/insertions in a non-repeat region or stop-loss variant] PP1: [Cosegregation with disease in multiple affected family members in a gene definitively known to cause disease]. PP2: Pathogenic supporting 2 [Missense variant in a gene that has a low rate of benign missense variation and in which missense variants are a common mechanism of disease]. PP3: Pathogenic supporting 3 [Multiple lines of computational evidence support a deleterious effect on the gene or gene product (conservation, evolutionary, splicing impact, etc.)]. PP4: Pathogenic supporting 4 [Patient’s phenotype or family history is highly specific for a disease with a single genetic etiology].

In two consanguineous families, i.e., RD024 and RP184, variants in *LCA5* were segregating with disease phenotype. The novel splice site variant, c.720+1G>T in family RD024, was found in the first nucleotide of intron 4, predicted to alter the correct splicing of the transcript (Figure 1 and Figure 3B). Splice AI predicted a loss of donor site (delta score of 0.98, 1 bp) which could cause a frameshift of *LCA5* in affected members of the family. Previously, a different splice site variant (c.720+1G>A) was reported at the same position in an LCA-affected family from a Spanish population [13], in which characterization of the functional impact of mutation using the mRNA from peripheral blood lymphocytes of homozygous, heterozygous carriers, and wild-type individuals followed by reverse transcription (RT) PCR revealed no RT-PCR products for the homozygous mutant individual. This result indicated no *LCA5* expression in the patient, suggesting the formation of an aberrant *LCA5* transcript because of the c.720+1G>A variant that seems to be sensitive to nonsense-mediated decay, further supporting the pathogenicity of the c.720+1G>T variant identified in family RD024 in the present study (Table 3). Further functional analysis is required to confirm the predicted effect of this variant on splicing. In family RP184, a homozygous nonsense variant of *LCA5* was detected, causing seven nucleotide deletion in c.1062_1068, resulting in the premature termination of protein p. (Tyr354*) (Supplementary Figures S2 and S6F). The variant is reported in ClinVar as pathogenic in association with the LCA phenotype (Table 3).

Two previously reported disease-causing single-nucleotide substitutions including c.316C>T and c.506G>A, causing p. (Arg106*) and p. (Arg169Gln) in the *RDH12* gene, were found segregating with the IRD phenotype in the homozygous state in the families RP002 and RP028, respectively (Table 2, Supplementary Figures S3 and S6D,E). Both variants are listed as pathogenic in ClinVar for LCA and are present in gnomAD (v2.1.1) with an allele frequency of 0.00001591 and 0.00001193, respectively.

*SPATA7* was mutated in two multigenerational families, i.e., RD027 and RP187. A c.864dup variant leading to a p. (Thr289Aspfs*3) was segregating in the RD027 family which had four affected cases in total (Supplementary Figures S4 and S6B). This variant is reported as pathogenic in ClinVar for LCA and had an allele frequency of 0.000007081 in gnomAD (v2.1.1). The proband of the RD027 family had congenital nyctalopia, nystagmus, and myopia (Table 1); however, other symptoms observed in different affected members include squint eyes (III.II and III.IV), hearing loss (III.V), intellectual disability (III.V), teeth (III.IV), and bone deformities (III.II and III.IV).

The other nonsense variant of *SPATA7,* i.e., c.1012C>T, causing a premature termination of protein at position 338 p. (Gln338*), was segregating in the RP187 family (Supplementary Figures S4 and S6G). The resulting chain termination causes a short protein p. (Gln338*).

Finally, the affected individuals of the families RP194, RP166, RP169, and RD036 were found to be homozygous for disease-causing variants in the *IFT140*, *LRAT*, *PRPH2,* and *TULP1* genes, respectively. Family RP194 was the consanguineous Punjabi family with six affected members in two generations (Supplementary Figure S2). Affected individuals carried a known homozygous missense variant c.998G>A in the *IFT140* gene, leading to an amino acid change from cysteine to tyrosine at the 333 position, i.e., p. (Cys333Tyr). This variant was segregating with disease phenotype (Supplementary Figure S6H) and had an allele frequency of 0.00001990 in gnomAD (v2.1.1). In ClinVar, it has been classified to be pathogenic and likely pathogenic for Saldino–Mainzer syndrome and retinitis pigmentosa phenotypes, respectively. A novel single-nucleotide substitution c.196G>C leading to missense variant p. (Gly66Arg) was identified in the *LRAT* gene in family RP166 (Figure 1 and Figure 3C). This family had three affected cases with congenital vision impairment, nyctalopia, oculodigital sign, and myopia in individuals (IV.II) (Table 1) with additional features of intellectual disability and epilepsy in other affected cases (IV.III). It is reported that the active site architecture of the LRAT protein is restrained by hydrogen bond interactions between histidine at position 60 and 71, and arginine at position 109; thus, conversion of a non-polar glycine to a polar residue, i.e., arginine, may impact the catalytic activity of protein [14].

A single in-frame variant in the current study with a deletion of six base pairs, i.e., c.620_625del, was detected in the *PRPH2* gene. This long novel deletion removes the amino acids aspartic acid and valine (p. (Asp207_Gly208del) in family RP169 (Figure 1 and Figure 3D). This in-frame deletion of two amino acids from the D2 loop of the interdiscal space of the PRPH2 protein, which is reported to harbor seventy percent of all known disease-causing mutations of *PRPH2,* may contribute to the structural modification of the variant protein, thus causing pathogenicity [15]. A canonical splice donor variant c.1495+1G>A in the *TULP1* gene was confirmed to segregate with disease phenotype in RD036. This variant is more likely to affect splicing by SpliceAI score for donor loss (delta score of 1.00, 1 bp) and donor gain (delta score of 0.98, −19 bp), thus causing frameshift. Previously, a G>C variant at the same position is reported in a non-syndromic RP-affected male individual [16]. The functional validation of the c.1495+1G>C variant has shown the skipping of exon 14, resulting in a frameshift with degradation of the mutant transcript by nonsense-mediated mRNA decay [17]. The variant c.1495+1G>A is listed as pathogenic in ClinVar for LCA 15, RP, and retinal dystrophy by multiple submitters and an allele frequency of 0.00001195 in gnomAD (v2.1.1) (Table 3 and Supplementary Figures S2 and S6C). A curated modified/new classification of previously reported likely pathogenic and novel variants based on the data of the present study is listed in Table 3.

## 4. Discussion

Next-generation sequencing has revolutionized diagnostic approaches to Mendelian disorders. The genetic screening of individuals with disease-causing variants in highly penetrant monogenic forms of IRDs is essential if genomic medicine is to fulfill its envisioned goals. Here, we report a total of 13 disease-causing variants in LCA-associated genes in a cohort of 15 multigeneration Pakistani families. Significant mutation heterogeneity exists within the population, considering that only the *CRB1*, *RDH12*, *LCA5,* and *SPATA7* genes were repeated in more than one family. Among 15 screened families, five families (33%) were segregating pathogenic variants in the *CRB1* (Crumbs homolog 1) gene, which encodes a transmembrane protein that localizes to the subapical region of Müller and photoreceptor cells [10,11]. Interestingly, we identified specified distribution patterns of *CRB1* mutations across different ethnicity groups. For example, a previously reported missense variant, i.e., p. Ser487Pro segregated with congenital onset of disease phenotype and severe loss of vision in three Pashtun families: RP159, RD023, and RP044 (Table 1). Previously, the first report on the p. Ser487Pro variant was in a sporadic male LCA/EOSRD patient of Pakistani descent who belonged to a consanguineous couple [18]. The same variant was also reported later in the Pashtun family segregating with the RP phenotype from the North-Western population of Pakistan [19,20]. Absence from gnomAD (v2.1.1), LOVD, and EXAC, presence in three Pashtun families (homogenous Pakistani subgroups) revealed in this study (Table 1), and previous reports from patients of Pakistani origin explains a possibility of a founder effect of the p. Ser487Pro variant in Pashtun ethnicity. In contrast, two Punjabi families (RD022 and RP196) segregated the same frame shift variant, i.e., p. (Leu1138Serfs*2) of *CRB1* gene. Previously, *CRB1* gene variants were reported to cause RP, LCA, cone-rod dystrophy and macular dystrophy phenotypes from different populations and are associated with characteristic fundus changes in the eye, particularly in the macula, often presenting with early-onset maculopathy and nummular pigmentation deposits, indicating significant damage to the retinal structure causing severe vision loss in affected individuals (Figure 2B and Supplementary Figure S5A) [11,12]. In this study, all *CRB1*-linked families were segregating the LCA phenotype (Table 1); however, variability in disease symptoms was present among the affected cases of these *CRB1*-linked families, even among those carrying the same disease-causing variant as listed in Table 1. The findings of *CRB1* gene variants and disease variability among patients are consistent with the results of a previously published study including a multinational cohort of patients [12].

We identified a homozygous pathogenic missense variant of a rarely mutated gene *IFT140*, c.998G>A causing p. (Cys333Tyr) in a single large Punjabi family, i.e., RP194 (Supplementary Figures S2 and S6H). Previously, Hull et al., 2016, reported this variant segregating with non-syndromic retinal dystrophy phenotype in two Punjabi families, one being from Pakistan and the other from Indian Punjab [21]. These data highlight the distribution of specific mutations across different ethnic groups in our population, urging extensive future studies using comprehensive molecular diagnostic approaches to reveal ethnicity-specific disease-causing variants for a better understanding of disease mechanisms. While some of the mutations are recurrent between patients from the same ethnicity, phenotypic heterogeneity can also be detected in cases carrying the same mutations [21]. Thus, in contrast to the early-onset LCA found in RP194 patients with p. (Cys333Tyr) mutation of the *IFT140* gene identified in this study, the patients reported by Hull et al., in 2016, carried the same mutation but manifested the disease after the first decade of life. Moreover, in the proband of RP194 (Table 1), additional symptoms of photophobia, myopia, heart and kidney diseases were also present. The presence of retinal dystrophy and chronic renal disease, the characteristic features of Mainzer–Saldino syndrome, reinforce monitoring of the other affected cases over time, as the IFT140 gene is reported to manifest both syndromic and non-syndromic phenotypes [21]. The same observation of phenotypic variability is also reported for the other ciliopathy genes [22,23].

In addition to this, pathogenic mutations in the *PRPH2* gene also lead to variable phenotypes including RP, cone dystrophy, cone-rod dystrophy, macular dystrophy, LCA, and retinopathies including pattern dystrophy, vitelliform macular dystrophy, and central areolar choroidal dystrophy (CACD), even in patients belonging to the same family [24,25,26]. In the present study, we identified a novel in-frame deletion of two amino acids, i.e., p. (Asp207_Gly208del) from the D2 loop of the PRPH2 protein, which is required for the maintenance of the flattened rim morphology of outer segments of photoreceptors [27]. Previously, a deletion of three amino acids at same position, i.e., p. (Asp207_Val209) is reported to cause highly variable autosomal dominant retinal dystrophy phenotypes within members of the same family [28]. Furthermore, deletion mutations involving p.207Asp and p.208Gly amino acids have been reported to cause autosomal dominant RP in cases from the USA, Canada, and Spain [29,30]. However, identification of the p. (Asp207_Gly208del) homozygous variant segregating with the LCA/EOSRD phenotype in four affected cases of the RP169 family supports the previous findings of association of homozygous PRPH2 variants with more severe phenotypes compared to heterozygous PRPH2 mutation carriers [31] (Figure 1, Figure 2 and Figure 3, Table 1 and Table 2). The heterozygous carriers of the p. (Asp207_Gly208del) variant, i.e., III.VI and III-VII of RP169 (Figure 1), are above 60 years of age and have a complaint of low vision from the last 4–5 years; however, IV.IV of RP169 is a asymptomatic carrier at the age of 36 years.

Previous studies have indicated that more than 90% of mutations in *SPATA7* are truncating alleles, either leading to frameshifts or nonsense codons [7,32,33], which is consistent with our results. We have identified a previously reported frame shift variant p. (Thr289Aspfs*3) of the *SPATA7* gene segregating with the LCA phenotype in the RD027 family. This variant is previously reported from different regions of the world including cases of Pakistani and mid-eastern origin [32,34,35]. Recently it has been found that the Spata7 protein is important not only in establishing a connecting cilium complex in photoreceptors, but also its maintenance through interactions with other ciliary proteins [33,36]. In conclusion, the five disease-causing variants identified in our study cohort are novel findings, highlighting the unique genetic spectrum of the Pakistani population. However, three previously reported variants, i.e., *LCA5*: c.1062_1068del, *TULP1*: c.1495+1G>A, and *RDH12*: c.316C>T, were identified for the first time in Pakistan. Furthermore, the use of consanguineous families with multiple affected cases helped us to classify novel identified variants, as well as previously reported likely pathogenic variants as pathogenic, as per the ACMG guidelines (Table 3). In this study, the main limitation/s to explaining genetic variants in phenotypic diversity is the unavailability of detailed clinical tests, including electroretinography and optical coherence tomography, due to unavailability of these diagnostic means. Furthermore, our results conclude that the CRB1 gene abnormalities are a common cause of LCA in our study cohort. However, we did not perform whole-genome sequencing and the families included in our study had severe cases of LCA with a strong family history. Nonetheless, this study contributes to a deeper comprehension of the severe retinal dystrophy phenotypes within the Pakistani genetic landscape and also carries practical implications for risk assessment, genetic counseling, and potential interventions to reduce overall burden of IRDs.

## Figures and Tables

**Table 1:** Demographic and clinical features of probands of 15 IRD families described in this study.

Sr. No.	Family ID	Proband ID	Age in Years at		Ethnicity	No. of Affected Cases in Family	Visual Acuity		Symptoms				
			Enrollment	Onset			OD	OS	Nyctalopia	Photophobia	Color Vision Problems	Hyperopia	Other/s
1	RD022	V.V	11	By birth	Punjabi	8	H.M	H.M	+	+	+	+	Oculo-digital sign
2	RD023	IV.III	12	One year	Pashtun	2	6/192 *	1/60 *	+	+	+	+	Nystagmus, HL, hypertension
3	RD024	IV.II	12	By birth	Saraiki	2	PL	PL	+	−	+	−	Nystagmus, Strabismus
4	RD027	III.II	20	By birth	Pothohari	4	6/60 *	6/60 *	+	−	−	−	Nystagmus, Myopia, Squint, BD
5	RD036	V.I	12	By birth	Pashtun	3	PL	PL	+	+	−	−	Nystagmus, Astigmatism
6	RP002	IV.VIII	48	By birth	Punjabi	8	NA	NA	+	−	−	−	-
7	RP028	IV.III	8	By birth	Punjabi	2	6/24 *	6/19 *	+	+	−	−	-
8	RP044	III.II	25	By birth	Pashtun	6	H.M	H.M	+	−	−	−	-
9	RP159	IV.IV	18	By birth	Pashtun	5	H.M	C.F	+	+	−	−	Nystagmus
10	RP166	IV.II	17	By birth	Pashtun	3	6/60 *	6/60 *	+	−	−	−	Oculo-digital sign, Myopia
11	RP169	IV.I	32	By birth	Punjabi	4	6/19 *	6/19 *	+	+	+	−	-
12	RP184	IV.VI	9	By birth	Punjabi	3	6/120 **	6/120 **	+	−	+	+	Nystagmus, teeth and BD, ID/MR
13	RP187	IV.I	15	By birth	Pashtun	5	PL	PL	+	−	−	−	-
14	RP194	IV.VIII	44	By birth	Punjabi	6	6/48 *	6/76 *	+	+	−	−	K and H problems
15	RP196	IV.II	18	By birth	Punjabi	2	C.F	C.F	+	+	−	−	Nystagmus, Myopia

Abbreviations: H.M: hand motion, C.F: counting fingers, PL: perception of light, NA: not available, HL: hearing loss, ID/MR: intellectual disability, BD: bone deformities, K and H: kidney and heart. The * symbol indicates the visual acuity was calculated using Early Treatment Diabetic Retinopathy Study (ETDRS) chart, the ** symbol indicates the visual acuity was measured using the Cardiff card.

**Table 2:** List of identified variants in Leber congenital amaurosis-associated genes in a Pakistani cohort of 15 consanguineous families.

Sr. No.	Family ID	Accession Number	Gene	Variant	Nucleotide Change	Protein Change	GnomAD	db SNP ID	Clin Var ID/Classification
1	RD022	NM_201253	*CRB1*	Chr1:197404401CTGTT>C	c.3411_3414del	p.(Leu1138Serfs*2)	NA	NA	NA
2	RD023	NM_201253	*CRB1*	Chr1:197390417T>C	c.1459T>C	p.(Ser487Pro)	NA	NA	2572562/likely pathogenic
3	RD024	NM_181714	*LCA5*	Chr6:80222928C>A	c.720+1G>T	p.?	NA	NA	NA
4	RD027	NM_018418.5	*SPATA7*	Chr14:88895738T>TA	c.864dup	p.(Thr289Aspfs*3)	0.000007081	rs386834241	1396/pathogenic
5	RD036	NM_003322	*TULP1*	Chr6:35467757C>T	c.1495+1G>A	p.?	0.00001195	rs281865168	99665/pathogenic
6	RP002	NM_152443	*RDH12*	Chr14:68191944C>T	c.316C>T	p.(Arg106*)	0.00001591	rs752242512	835782/pathogenic
7	RP028	NM_152443	*RDH12*	Chr14:68193755G>A	c.506G>A	p.(Arg169Gln)	0.00001193	rs971610277	623219/pathogenic
8	RP044	NM_201253	*CRB1*	Chr1:197390417T>C	c.1459T>C	p.(Ser487Pro)	NA	NA	2572562/likely pathogenic
				Chr1:197390892AC>A	c.1935delC	p.(Asp645Glufs*20)	NA	NA	NA
9	RP159	NM_201253	*CRB1*	Chr1:197390417T>C	c.1459T>C	p.(Ser487Pro)	NA	NA	2572562/likely pathogenic
10	RP166	NM_004744	*LRAT*	Chr4:155665674G>C	c.196G>C	p.(Gly66Arg)	NA	NA	NA
11	RP169	NM_000322	*PRPH2*	Chr6:42672305ACGCCGT>A	c.620_625del	p.(Asp207_Gly208del)	NA	NA	NA
12	RP184	NM_181714	*LCA5*	Chr6:80201334TGTTTTCG>T	c.1062_1068del	p.(Tyr354*)	NA	rs1769845495	810630/pathogenic
13	RP187	NM_018418	*SPATA7*	Chr14:88895791C>T	c.1012C>T	p.(Gln338*)	0.000003986	rs1188647815	NA
14	RP194	NM_014714	*IFT140*	Chr16:1637210C>T	c.998G>A	p.(Cys333Tyr)	0.00001990	rs773372123	438181/pathogenic
15	RP196	NM_201253	*CRB1*	Chr1:197404401CTGTT>C	c.3411_3414del	p.(Leu1138Serfs*2)	NA	NA	NA

**Table 3:** Curated variant classification of novel and previously reported likely pathogenic variants based on data of present study as per ACMG guidelines.

Sr. No.	Gene	Nucleotide Change	Clin Var ID/Classification	Variant Interpretation (Codes Met)	Curated Variant Classification
1	*CRB1*	c.3411_3414del	NA	PM2_supporting, PVS1, PP4, PP1, PP3	pathogenic
2	*CRB1*	c.1459T>C	2572562/likely pathogenic	PM2_supporting, PP3, PP4, PP1	pathogenic
3	CRB1	c.1935delC	NA	PM2_supporting, PVS1, PP4, PP1, PP3	pathogenic
4	*LCA5*	c.720+1G>T	NA	PM2_supporting, PVS1, PP4, PP1, PP3	pathogenic
5	*LRAT*	c.196G>C	NA	PM2_supporting, PP3, PP4, PP1, PP2	pathogenic
6	*PRPH2*	c.620_625del	NA	PM2_supporting, PP4, PP1, PM4	pathogenic
7	*SPATA7*	c.1012C>T	NA	PM2_supporting, PVS1, PP4, PP1, PP3	pathogenic

## Data Availability

The original contributions presented in this study are included in the article/Supplementary Materials. Further inquiries can be directed to the corresponding authors.

## Supplementary Materials

The following supporting information can be downloaded at https://www.mdpi.com/article/10.3390/genes15121646/s1, Figure S1: Pedigrees of two consanguineous families (RD023 and RP159) in which reported variants in *CRB1* gene were identified. Empty squares and circles show the unaffected males and females, respectively. The filled shapes show the affected individuals. The symbol labeled with a red arrow in each pedigree highlights the proband. Consanguineous unions are indicated by double lines. Figure S2: Pedigrees of three inbred families RD036, RP184, and RP194 in which reported variants in the *TULP1*, *LCA5*, and *IFT140* genes were identified, respectively. Empty squares and circles show the unaffected males and females, respectively. The filled shapes show the affected individuals. The symbol labeled with a red arrow in each pedigree highlights the proband. Consanguineous unions are indicated by double lines. Figure S3: Pedigrees of two consanguineous families (RP002 and RP028) in which reported variants in the *RDH12* gene were identified. Empty squares and circles show the unaffected males and females, respectively. The filled shapes show the affected individuals. The symbol labeled with a red arrow in each pedigree highlights the proband. Consanguineous unions are indicated by double lines. Figure S4: Pedigrees of two consanguineous families (RD027 and RP187) in which reported variants in the *SPATA7* gene were identified. Empty squares and circles show the unaffected males and females, respectively. The filled shapes show the affected individuals. The symbol labeled with a red arrow in each pedigree highlights the proband. Consanguineous unions are indicated by double lines. Figure S5: Fundus photographs of left and right eye of proband of family RD023 (A), RD027 (B), RP159 (C) and RP194 (D). Figure S6: Sequence chromatogram of families with reported variants in LCA-associated genes (A–H). The wild type sequences are given on the left side and the variant sequences are given on the right side. Table S1: Variants interpretation data.
