# Supplementary figures and images for: Phenotypic and Genetic Heterogeneity of a Pakistani Cohort of 15 Consanguineous Families Segregating Variants in Leber Congenital Amaurosis-Associated Genes
Source: Genes (Basel). 2024 Dec 21;15(12):1646. doi: 10.3390/genes15121646 (PMC11728111; doi:10.3390/genes15121646)

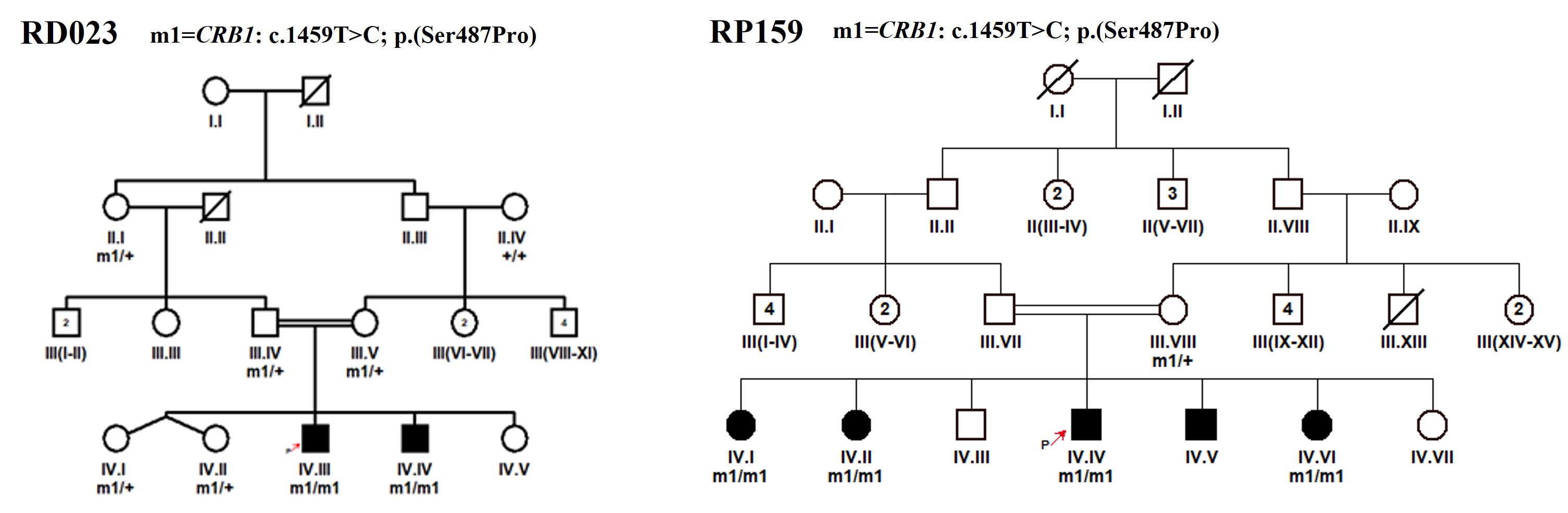

Supplement: Supplementary file 1 [file genes-15-01646-s001.zip › genes-3342075-supplementary Figure S1.jpg]

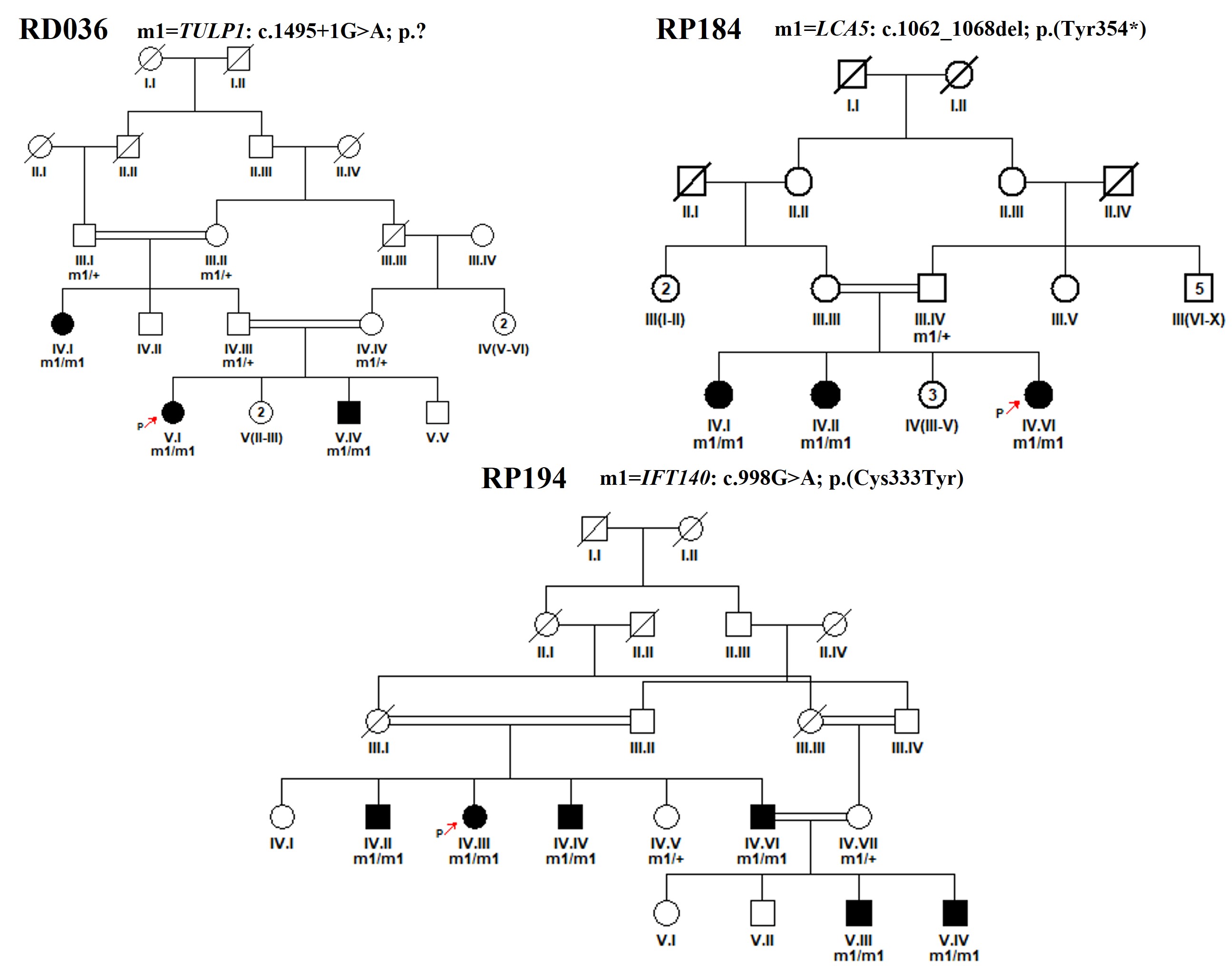

Supplement: Supplementary file 1 [file genes-15-01646-s001.zip › genes-3342075-supplementary Figure S2.jpg]

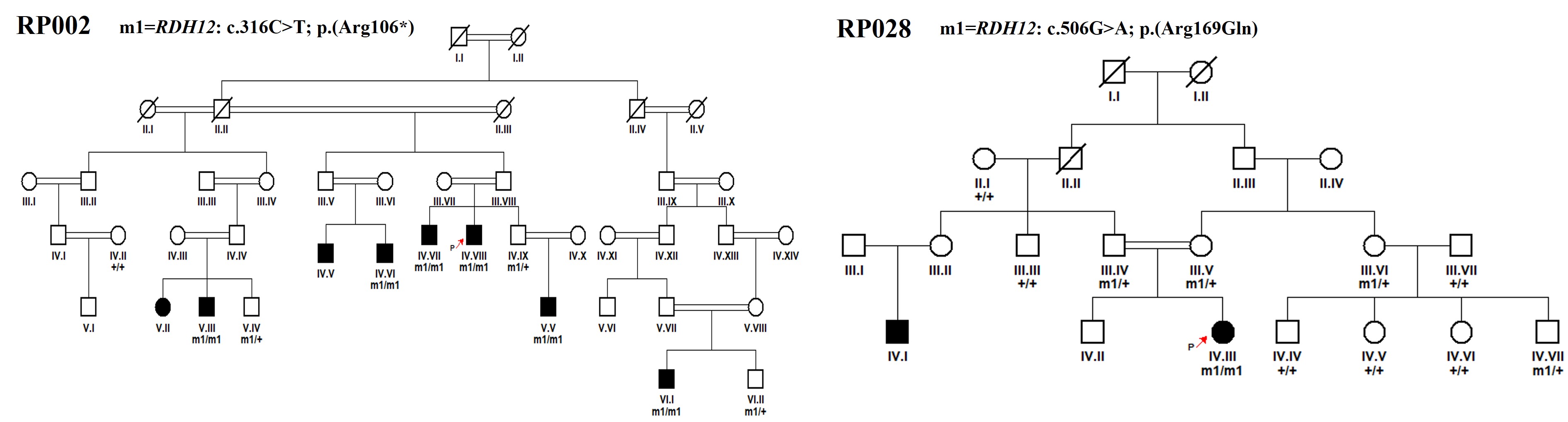

Supplement: Supplementary file 1 [file genes-15-01646-s001.zip › genes-3342075-supplementary Figure S3.jpg]

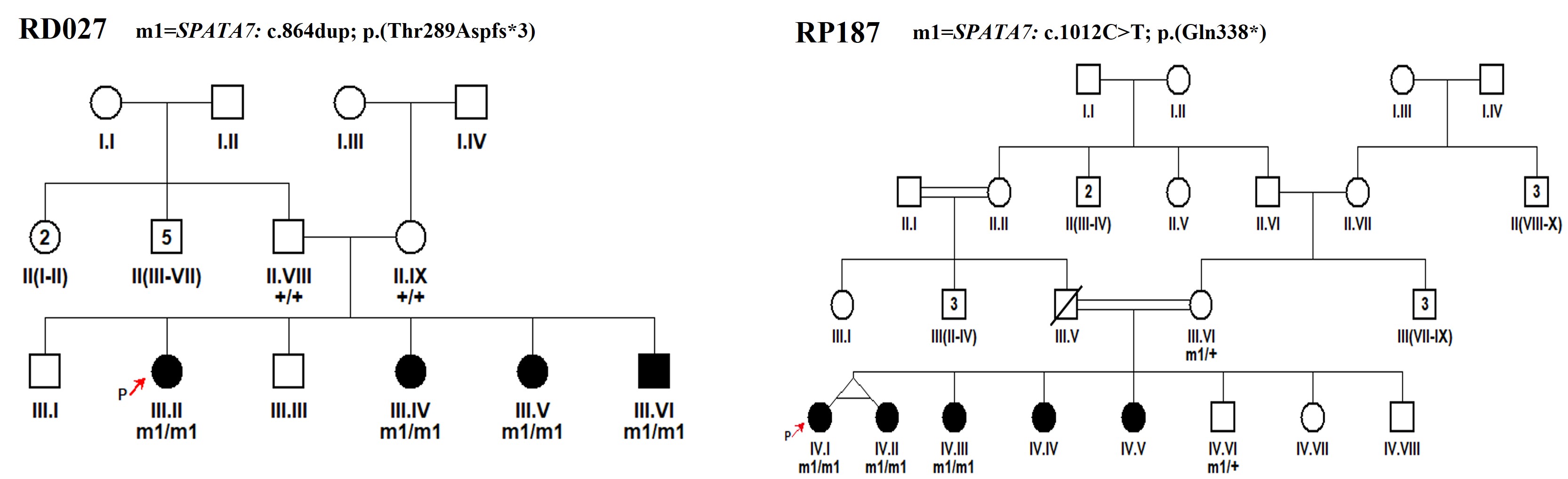

Supplement: Supplementary file 1 [file genes-15-01646-s001.zip › genes-3342075-supplementary Figure S4.jpg]

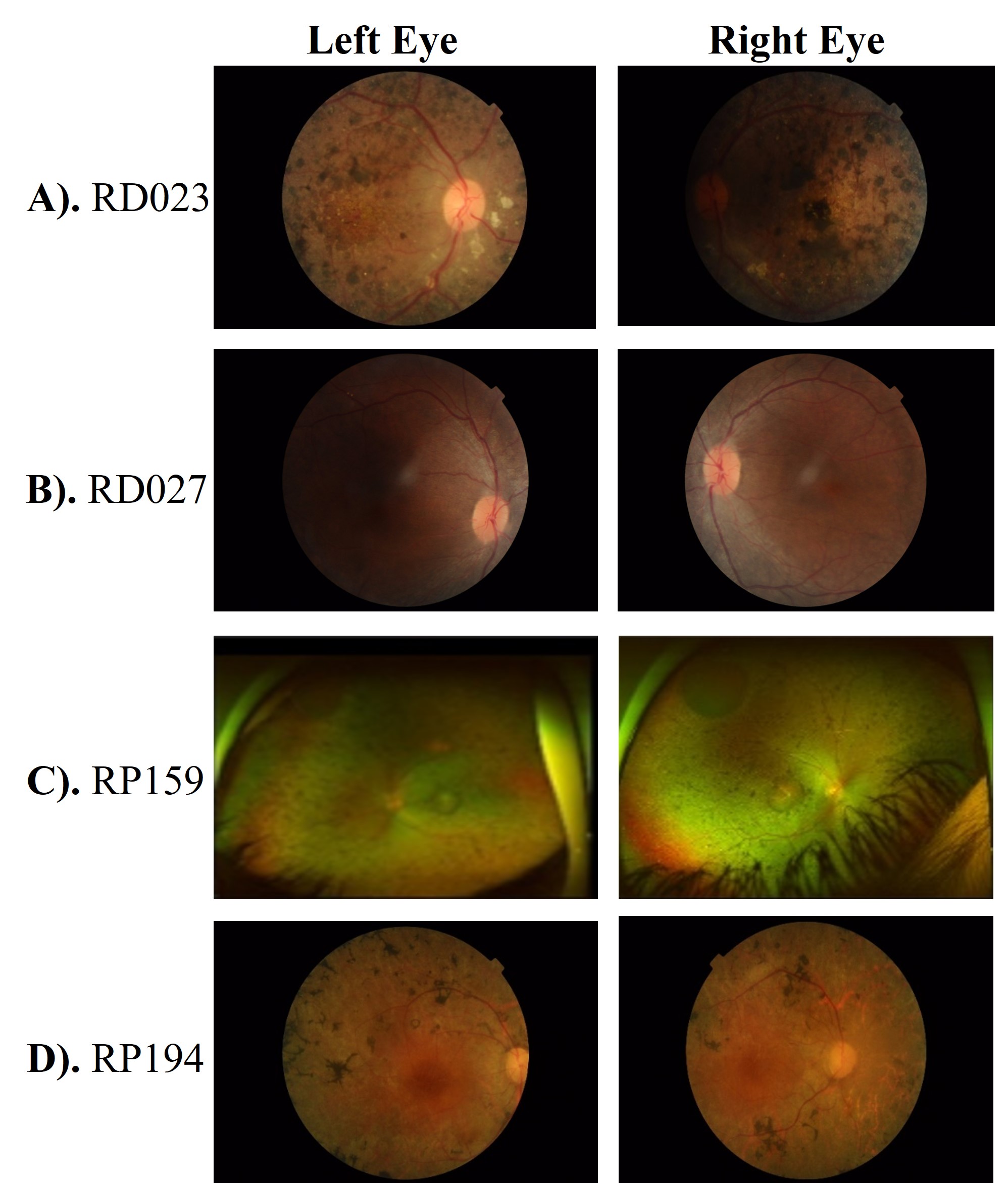

Supplement: Supplementary file 1 [file genes-15-01646-s001.zip › genes-3342075-supplementary Figure S5.jpg]

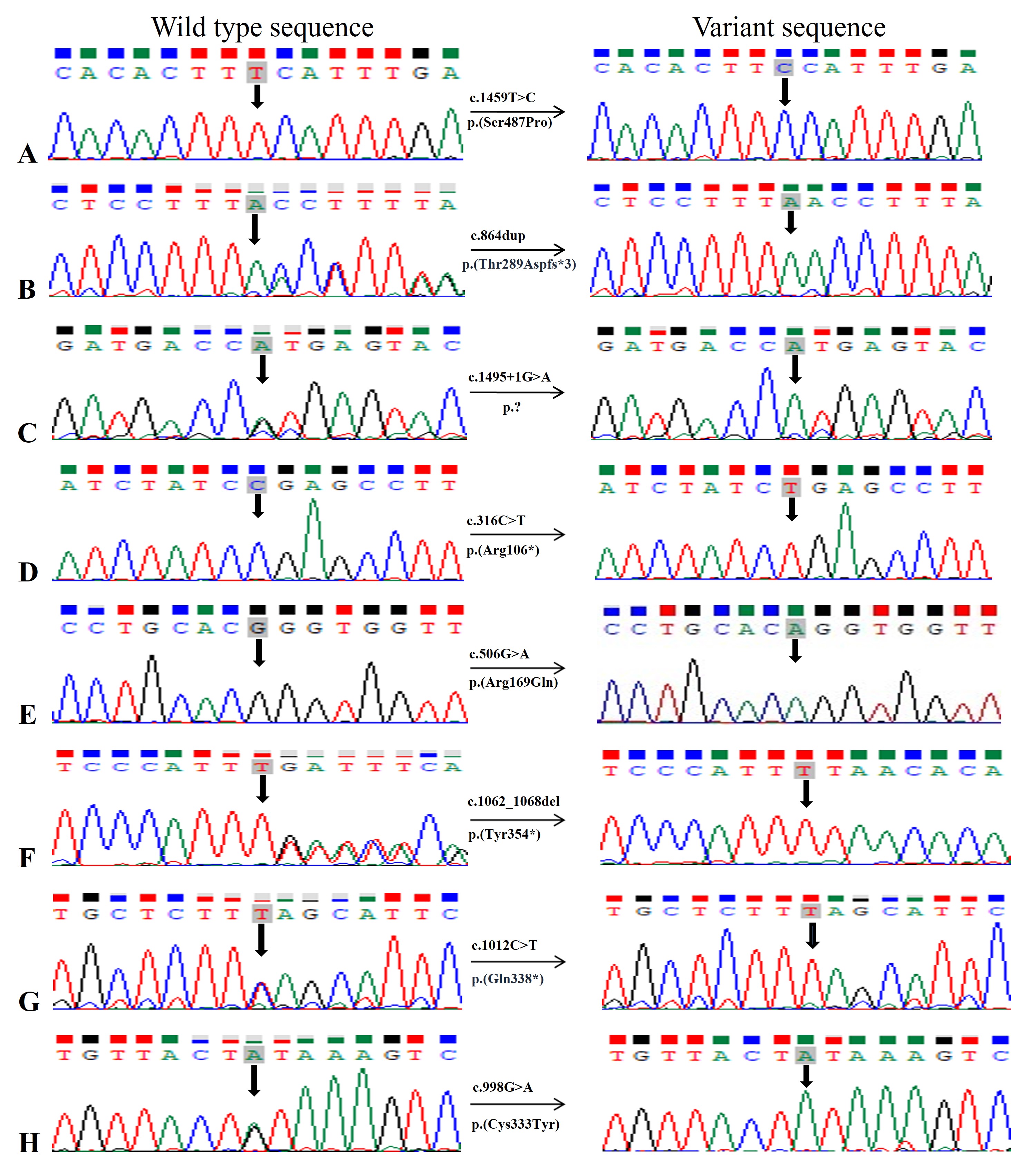

Supplement: Supplementary file 1 [file genes-15-01646-s001.zip › genes-3342075-supplementary Figure S6.jpg]
